# Obesity and Metabolic Syndrome in the 21st Century: A Narrative Review of Cardiometabolic Risk and Global Policy Response

**DOI:** 10.7759/cureus.97204

**Published:** 2025-11-19

**Authors:** Daniel Sanchez, Esaúl Marroquín León, Celina N Angulo Salgado, Yareli Santos Martinez, Perla Delgado Figueroa, Marion Camacho Escárcega, Alexa Herrera Cohen, Xavier Antonio Sanchez Garcia, Jorge Alberto Riancho Guzman, Sayra E Rincón Haro

**Affiliations:** 1 Medicine, Universidad Westhill, Mexico City, MEX; 2 Internal Medicine, Universidad Westhill, Mexico City, MEX; 3 General Medicine, Universidad del Valle de México, Hermosillo, MEX; 4 Research, Universidad Westhill, Mexico City, MEX; 5 Pediatrics, Universidad La Salle, Mexico City, MEX; 6 Plastic and Reconstructive Surgery, Hospital Angeles Pedregal, Mexico City, MEX; 7 Plastic and Reconstructive Surgery, Hospital Central Militar, Mexico City, MEX; 8 Laparoscopic Surgery, Hospital Angeles Pedregal, Mexico City, MEX; 9 Research, Universidad La Salle, Mexico City, MEX

**Keywords:** cardiovascular disease, dyslipidemia, insulin resistance, metabolic syndrome, obesity, public health policy

## Abstract

The global rise of cardiovascular disease stems from the combined effects of metabolic syndrome and obesity. The clinical picture of these diseases develops through complex interactions between genetic and environmental factors and lifestyle elements which generate central obesity, insulin resistance, dyslipidemia, arterial hypertension, and systemic inflammation leading to a prothrombotic, proatherogenic, and pro-inflammatory state that increases the risk of acute coronary syndrome, acute myocardial infarction, cerebrovascular diseases, heart failure, and non-alcoholic fatty liver disease which occur at younger ages with distinct clinical patterns and severe cardiometabolic effects. The worldwide occurrence of these conditions has risen dramatically to become a worldwide pandemic which demands immediate public health action for effective policy implementation. The existing literature about this topic requires synthesis to effectively evaluate the evidence and recommendations regarding obesity and metabolic syndrome pathophysiology, clinical manifestations, cardiovascular effects, and public health interventions and their outcomes to determine which policies succeeded and which areas need improvement since taxes on high-calorie products and front-of-package labeling have demonstrated promising outcomes.

## Introduction and background

A pandemic is defined as "an epidemic occurring over a very wide area, crossing international boundaries, and usually affecting a large number of people" [1]. Rates of overweight and obesity increased at the global and regional levels, and in all nations, between 1990 and 2021. In 2021, an estimated one billion adult males and 1.11 billion adult females were overweight and obese. Countries with low and middle incomes, particularly regions such as North Africa, Asia, the Middle East, and sub-Saharan Africa, have experienced the fastest increases in obesity prevalence. Based on the expected persistence of previous trends, it is projected that, by 2025, the total of adults with overweight and obesity will reach 3.8 billion, which will constitute over half of the estimated global adult population at the time [2,3].

Obesity is a medical condition characterized by the excessive accumulation of body fat, which poses a health risk. The World Health Organization (WHO) defines obesity as a BMI of 30 kg/m² or more. Aside from BMI, central obesity is an important predictor of health risk, as abdominal fat is connected to metabolic and cardiovascular issues [4]. 

Cardiovascular risk refers to the probability of an individual developing cardiovascular disease (CVD), including coronary artery disease (CAD), stroke, and related conditions. Obesity and metabolic syndrome (MetS) significantly elevate the potential of subsequent disease, through their link to various cardiovascular risk factors [2,4]. CVDs, such as coronary heart disease or cerebrovascular accident, frequently exhibit a number of aetiologically associated cardiometabolic risk factors, including dyslipidemia, hypertension, and elevated fasting plasma glucose, which may or may not coexist with multiple inflammatory markers (e.g., C-reactive protein (CRP), uric acid, and cytokines) and a prothrombotic state (e.g., plasminogen activator inhibitor-1) [5].

The deep interlink between both pathologies and, in most cases, the subsequent cardiometabolic risks is why it is important to understand what each pathology represents on more than an individual scale, as well as its implications for global health. Comprehending the relationship between these three big public health issues and their interaction may help pave the way towards more efficient prevention strategies, better treatment outcomes, and an overall improvement in quality of life [6,7].

## Review

Pathophysiology of cardiometabolic risk in obesity and MetS

Insulin Resistance 

Insulin resistance functions as the main factor which creates cardiometabolic risk in obese patients and those with MetS. The body uses insulin to help glucose enter skeletal muscles and fat cells while it blocks gluconeogenesis in the liver. The development of insulin resistance disrupts these processes which results in hyperinsulinemia and dysglycemia and multiple metabolic problems [8]. 

The process of lipolysis that breaks down excess fat cells produces high levels of free fatty acids (FFAs) which accumulate in non-fat tissues, including the liver and muscle cells, to interfere with insulin signaling pathways. The process happens through JNK activation which leads to IRS-1 serine/threonine phosphorylation that blocks PI3K-Akt pathway activation and decreases GLUT4-mediated glucose transport [9,10]. Studies conducted on mice have shown that JNK1 deficiency in fat tissue helps protect against insulin resistance development when mice consume high-fat diets [10]. 

The liver experiences insulin resistance which leads to de novo lipogenesis and elevated very-low-density lipoprotein (VLDL) secretion and results in metabolic dysfunction-associated steatotic liver disease (MASLD) that worsens systemic lipid disorders [11]. The condition known as atherogenic dyslipidemia develops when insulin resistance causes high triglyceride levels and small dense low-density lipoprotein (LDL) particles and low high-density lipoprotein (HDL) levels which together raise the risk of atherosclerotic cardiovascular disease (ASCVD) [12]. 

The body experiences chronic low-grade inflammation because of TNF-α and IL-6 cytokines released by adipose tissue which disrupts insulin signaling and damages endothelial function [13]. The pathogenesis of CVD in obesity and MetS depends on insulin resistance because it creates a network between glucose dysregulation and lipid abnormalities and inflammation and endothelial dysfunction at both cellular and systemic levels [8-12].

Atherogenic Dyslipidemia

Building upon the metabolic disturbances driven by insulin resistance, atherogenic dyslipidemia represents the lipid manifestation of the MetS. The condition presents with three primary features that include elevated triglycerides and decreased high-density lipoprotein cholesterol (HDL-C) and the presence of small dense LDL particles. The profile shows a high risk of developing ASCVD which can occur with typical low-density lipoprotein cholesterol (LDL-C) levels [14,15].

Pathophysiological Mechanisms 

In insulin-resistant states, the liver produces more VLDL through excessive FFA metabolism [16]. The body activates cholesteryl ester transfer protein (CETP) more when triglyceride levels rise which results in triglyceride storage within LDL and HDL particles. The triglyceride-rich lipoproteins undergo hepatic lipase hydrolysis which produces small dense LDL and dysfunctional HDL particles [17]. 

The study by Tribble et al. demonstrates that small dense LDL particles exhibit greater atherogenic potential than large buoyant LDL because they tend to become oxidized more easily [18], penetrate the arterial intima more efficiently, and have a lower affinity for LDL receptors, prolonging their circulation [19]. The reduction of HDL-C levels creates an environment that worsens atherosclerosis because it disrupts the process of reverse cholesterol transport [20]. 

Clinical Implications 

The Quebec Cardiovascular Study established small dense LDL as an independent risk factor for ischemic heart disease because men with the highest small dense LDL levels experienced three times greater risk than men with larger LDL particles [21]. The triglyceride-to-HDL-C ratio functions as a powerful indicator of insulin resistance in patients with type 2 diabetes and MetS and shows a strong ability to predict cardiovascular events [22].

Therapeutic Perspectives 

The fundamental basis of management consists of diet and exercise modifications which enhance lipid metabolism and insulin sensitivity. The medication effectively lowers LDL-C levels, but it does not affect small dense LDL or hypertriglyceridemia according to Jun et al. [23]. The combination of fibrates with omega-3 fatty acids leads to triglyceride reduction and small HDL-C elevation, but research indicates no substantial cardiovascular advantages [23].

The present therapeutic methods work to manage every risk factor which doctors can modify. PCSK9 inhibitors reduce LDL-C and ASCVD events, but they do not impact triglyceride or HDL-C levels [24]. The REDUCE-IT trial showed that high-dose icosapent ethyl (EPA) cut major cardiovascular events by 25% in patients with hypertriglyceridemia who were already on statins [25]. ApoC-III and ANGPTL3 inhibitors have emerged as novel therapeutic agents which show potential to decrease triglycerides and VLDL production [26].

Atherogenic dyslipidemia exists as a dual-purpose condition because it demonstrates metabolic disorder symptoms while simultaneously creating conditions that result in atherosclerosis development. The recognition highlights the requirement for treatment methods which extend past LDL-C reduction because they need to handle triglycerides and small dense LDL and improve HDL function to reduce cardiovascular risk effectively [24-26]. 

Systemic Inflammation and Endothelial Dysfunction

Systemic inflammation at low levels creates the link between obesity and MetS and CVD development. Visceral fat functions as an active endocrine organ which produces pro-inflammatory cytokines including tumor necrosis factor-alpha (TNF-α) and interleukin-6 (IL-6) and resistin while reducing the protective adipokine production of adiponectin to create conditions for insulin resistance and accelerated atherogenesis and endothelial dysfunction [27,28]. The growth of fat tissue leads to macrophage entry and NF-κB pathway activation which creates a continuous inflammatory process [29]. The elevation of IL-6 leads to the increased hepatic production of CRP which medical science has established as a validated cardiovascular risk indicator. The presence of TNF-α leads to two main effects on blood vessels: it damages insulin receptors and reduces nitric oxide (NO) levels and creates oxidative stress which causes blood vessels to stiffen and lose their ability to relax [30,31]. 

The first sign of atherosclerosis development appears as endothelial dysfunction which results from an imbalance between vasodilatory and vasoconstrictive factors and elevated oxidative stress and increased expression of adhesion molecules including VCAM-1, ICAM-1, and E-selectin. The dysfunctional state enables leukocytes to stick to vessel walls while moving through the intima which leads to plaque development and growth [32,33]. Research studies about future developments demonstrate how biological processes shape medical practice. The Women's Health Study established that high-sensitivity CRP (hs-CRP) levels above the normal range are directly linked to heart attacks and strokes while ignoring LDL-C measurements [34]. The Multi-Ethnic Study of Atherosclerosis (MESA) showed that subclinical atherosclerosis and future cardiovascular events could be predicted through the combination of inflammatory markers and impaired endothelial function [35]. The brachial artery flow-mediated dilation (FMD) of patients with obesity and MetS demonstrates decreased values which function as a surrogate indicator of endothelial dysfunction and strongly indicate cardiovascular risk [36]. 

Weight reduction and regular physical activity function as lifestyle changes which decrease body-wide inflammation and enhance blood vessel function. The combination of these interventions results in superior outcomes through pharmacological methods because statins lower cholesterol levels and simultaneously decrease CRP and directly combat vascular inflammation [37]. Research into targeted immunomodulation has become a major focus for scientists throughout the previous few years. The CANTOS trial proved that IL-1β inhibition through canakinumab treatment decreased cardiovascular event rates while leaving lipid profiles unchanged which established inflammation as the primary cause of atherosclerosis [38]. The pleiotropic effects of glucose-lowering drugs GLP-1 receptor agonists and SGLT2 inhibitors lead to decreased inflammation and better endothelial function which indicates their protective effects on the heart beyond blood sugar management [39]. 

Systemic inflammation, together with endothelial dysfunction, acts as essential links which lead metabolic problems to develop into full CVD. The fast progression of atherosclerosis occurs because of these factors which demonstrates that treating both metabolic and inflammatory pathways is essential for lowering current cardiovascular risk. 

Hypertension and Neurohormonal Dysregulation

Hypertension stands as a major dangerous condition of the MetS which develops through neurohormonal system imbalances that occur in obesity and insulin resistance. The body activates the renin-angiotensin-aldosterone system (RAAS) and sympathetic nervous system (SNS) when excess body fat exists because these systems lead to blood vessel tightening and salt conservation and blood vessel structural changes [40,41]. The production of angiotensinogen by adipose tissue results in elevated angiotensin II concentrations both within the tissue and throughout the body which causes aldosterone production to rise and vascular wall oxidative stress to increase [42]. The two distinct effects of hyperinsulinemia operate together to produce worsening vascular function. The condition leads to SNS activation which results in increased sodium reabsorption by the kidneys and simultaneously causes damage to endothelial NO production [43,44]. 

Hypertension causes endothelial dysfunction through reduced NO bioavailability and elevated endothelin-1 production and increased vascular oxidative stress which results in faster arterial stiffening and left ventricular hypertrophy and diastolic dysfunction [45,46]. The RAAS and SNS systems experience increased activation because of systemic inflammation and adipokine imbalance which leads to a dangerous cycle between metabolic and hemodynamic issues [47]. Research studies have shown that obese people with high blood pressure have higher levels of plasma renin activity and aldosterone and sympathetic nerve activity than normal-weight people who have the same blood pressure levels [48]. 

The therapeutic methods which decrease body weight and enhance insulin sensitivity work to decrease neurohormonal system overactivity. The RAAS can be blocked through pharmacological means using angiotensin-converting enzyme inhibitors (ACEIs) and angiotensin receptor blockers (ARBs) and mineralocorticoid receptor antagonists (MRAs) which decrease blood pressure while enhancing vascular and metabolic health [49,50]. Likewise, sympatholytic strategies, though less commonly employed, demonstrate that inhibition of adrenergic overdrive ameliorates insulin resistance and vascular inflammation [51]. The SGLT2 inhibitors and GLP-1 receptor agonists generate various beneficial effects which decrease blood pressure while creating positive neurohormonal changes that benefit the entire cardiometabolic system [52,53]. 

Hypertension within MetS exists as a multifaceted condition which extends beyond blood pressure control because it stems from neurohormonal system imbalances. The diagnosis of this condition shows that treatment methods need to address cardiovascular stress and blood vessel issues and metabolic-inflammatory conditions to provide complete heart protection.

Clinical manifestations and major cardiovascular outcomes

Coronary Artery Disease (CAD) and Acute Myocardial Infarction (AMI)

The 21st century has seen CAD and AMI emerge as major cardiovascular problems because obesity and MetS create conditions that lead to endothelial damage, atherosclerosis, and thrombosis through inflammatory responses, metabolic problems, and oxidative stress [54-60]. The worldwide obesity epidemic, which affects more than 25% of middle-income nations, has made obesity the leading risk factor for CAD when BMI values are above 30 kg/m² and waist circumference increases are directly linked to a 40% higher CAD risk with each 10 cm increase (OR 1.04; 95% CI 1.01-1.07; p=0.013) [54,56,57,59,60]. The risk of CVD becomes more severe in MetS, defined by conditions including hypertension, dyslipidemia, hyperglycemia, and central obesity, which increases CVD risk by two times and type 2 diabetes mellitus (T2DM) risk by five times and creates the early development of CAD through pathways like insulin resistance and vascular inflammation mediated by adipocytokines [54-56,59,60]. 

The pathophysiological process of excess body fat or adiposity creates oxidative stress through reactive oxygen species (ROS) production from damaged adipose tissue which results in decreased NO availability, blood vessel constriction, and unstable plaques that leads to a higher risk of AMI [54,56]. The presence of pro-inflammatory cytokines such as IL-6 and TNF-α from adipose tissue accelerates the process of endothelial damage and atherosclerosis development; the combination of low HDL-C levels (<40/50 mg/dL) with elevated fasting plasma glucose (FPG >100 mg/dL) in MetS patients creates a synergistic effect that increases CAD risk through a dose-dependent relationship (OR values from 3.72 to 4.09 for 2-5 components) [54,56,59,60]. Even in metabolically healthy obesity (MHO) and overweight (MHOW) individuals without MetS criteria, the inherent adiposity leads to subclinical inflammation which contradicts the "obesity paradox" that shows higher BMI (>25 kg/m²) might protect against CVD events in certain situations but actually can lead to increased CVD events [54-56,60]. 

Research outcomes based on large population studies and meta-analyses show that MetS in obese patients leads to higher CAD and AMI risks with AMI hazard ratios of 1.68 (95% CI 1.27-2.22) in obese patients and 1.58 (95% CI 1.13-2.21) in overweight patients regardless of their nationality [57-60]. The 30-day risk of AMI and long-term mortality rates are higher in patients with MetS who underwent percutaneous coronary intervention (PCI) (34% of patients in this setting), the 30-day AMI risk is 2.2% in patients with MetS compared to 1.8% in patients without MetS (p=0.013), and the long-term mortality risk is 24% in patients with MetS compared to 19% in patients without MetS (p<0.001). The relationship between MetS and mortality risk becomes less significant after controlling for comorbidities like diabetes (HR 0.95; 95% CI 0.86-1.05; p=0.35) which shows this condition has the strongest impact as a prognostic factor [55,58,59]. The results of multiple meta-analyses show that MetS results in elevated death rates (all-cause mortality RR 1.22; 95% CI 1.10-1.35), cardiovascular death (RR 1.36; 95% CI 1.15-1.61), and recurrent myocardial infarction (MI) (RR 1.46; 95% CI 1.24-1.72), with low HDL-C and hyperglycemia as key drivers [58-60]. 

The "obesity paradox", as a predictor of outcomes, emerge variably: non-diabetic obese patients with MetS show better long-term survival rates due to their metabolic reserves, but MHO/MHOW patients still face higher CVD risk (RR 1.58 for MHO (95% CI 1.34-1.85); RR 1.34 for MHOW (95% CI 1.23-1.46)) according to studies [55,60]. In patients with established CVD, components of MetS, particularly hypertension (>130/85 mmHg) and dyslipidemia, further worsen clinical outcomes. When combined with oxidative stress arising from excess adiposity, these factors accelerate plaque instability, rupture, and thrombosis, ultimately increasing the risk of AMI, which commonly manifests as an acute clinical event [54,56,58].

The integrated evidence shows that specific interventions need to be developed to reduce CAD and AMI risks in obesity and MetS patients with particular goals including MetS component early screening for high-risk patients who have two or more factors to start lifestyle changes, antioxidant treatments, and glycemic control to decrease oxidative stress and achieve FPG levels <100 mg/dL for risk reduction of all-cause and cardiovascular death, the need to separate diabetic from non-diabetic patients addressing individualized post-PCI care, and the need for worldwide weight management strategies to stop MHO patients from developing unhealthy habits in order to reduce CVD risk by 50% in high-risk populations through diverse prevention approaches [54-60].

Cerebrovascular Disease and ⁠Stroke

Cerebrovascular disease, including cerebral small vessel disease (CSVD) and stroke, has become a prominent cardiometabolic complication affecting patients with obesity and MetS during the 21st century; this condition causes microvascular damage to cerebral arterioles, capillaries, and venules which leads to lacunar infarcts, white matter hyperintensities (WMH), microbleeds, and brain atrophy [61-64]. The components of MetS, including central obesity, dyslipidemia, hypertension, insulin resistance, and hyperglycemia, tend to worsen stroke conditions which primarily consist of atherothrombotic ischemic and hemorrhagic types [64-67]. The global obesity epidemic affects 650 million adults, while MetS creates chronic low-grade inflammation and damages blood vessel linings which worsens cerebrovascular disease in subclinical patients where early intervention is effective leading to better outcomes [62-64,66-68].

The pathogenic mechanisms behind these associations involve chronic inflammation with the activation of adipose tissue-derived cytokines like TNF-α and IL-6 impairing endothelial NO availability while producing oxidative stress and resulting in blood-brain barrier damage [61,64,69]. The core feature of MetS hypertension functions as an independent risk factor for developing CSVD, but dyslipidemia and diabetes accelerate WMH progression and create unstable vascular plaques [61,64,69]. The preclinical models of middle cerebral artery occlusion (MCAO) and stroke-prone spontaneously hypertensive rats (SHR/SP) demonstrate how MetS causes neuroglial inflammation through the activation of thioredoxin-interacting protein (TXNIP) and NOD-like receptor protein 3 (NLRP3) inflammasome which results in hippocampal and prefrontal cortical neuronal dysfunction [69].

MRI neuroimaging research has identified particular structural changes in patients undergoing obesity through their reduced prefrontal cortex, orbitofrontal cortex, precentral gyrus, limbic system gray matter volume, and frontal and temporal lobe cortical thinning [63]. White matter integrity shows damage through demyelination and lower fractional anisotropy values in brain tracts including the corpus callosum, corticospinal tract, thalamic radiations, corona radiata, and superior longitudinal fasciculus (SLF) [63]. The changes become permanent after adulthood, but weight control during early adulthood can prevent them from occurring. MRI serves as a biomarker for obesity-related cerebrovascular pathology because South Asians develop visceral adiposity which makes them more susceptible to these changes [63].

The clinical evidence shows that MetS increases stroke risk as demonstrated in prospective cohort studies where total stroke odds ratios are between 1.48 and 1.89 when using the National Cholesterol Education Program Adult Treatment Panel III (NCEP-ATP III), International Diabetes Federation (IDF), and ethnic-specific IDF criteria with moderate diagnostic accuracy (AUC-ROC 0.78-0.79) [65,66]. The study of a hypertensive patient cohort showed that participants who had high variability in three or more MetS parameters evaluated using measures such as coefficient of variation, standard deviation, average real variability, and variability independent of the mean developed higher risks for total stroke (HR 1.29) and ischemic stroke (HR 1.31) than the cohort without high-variability parameters; the relationship was mainly caused by systolic blood pressure and HDL-cholesterol variability [67].** **Cerebral small vessel disease is highly prevalent in adults over 50 years, with prevalence ranging from 14.5% for lacunar lesions and 65.4% for WMH. Additionally, MetS worsens these conditions with a relative risk of 2.14-2.83 in subclinical settings, also unveiling a higher risk for Alzheimer's disease and related dementias among women [61,62].

The "obesity paradox" is observed after CVD incidents in MHO (lacking dyslipidemia, diabetes, or hypertension) experience lower hazard rates (0.76-0.85) for stroke recurrence, peripheral vascular disease, and CVD death than normal BMI patients with no risk factors; the combination of multiple metabolic risks and elevated BMI increases morbidity including non-fatal coronary heart disease and heart failure [68]. Cognitive impairment and vascular dementia cause major problems in patients with midlife MetS leading to increased WMH burden, gray matter atrophy, and executive function decline, memory, attention, visuoconstructive deficits, and language impairment although results vary because of heterogeneous MetS criteria and cognitive assessment methods [62,64,69,70]. 

Heart Failure (HF)

HF represents one of the most severe cardiovascular consequences of obesity and MetS, arising from the convergence of hemodynamic overload, systemic inflammation, and neurohormonal dysregulation. Excess adiposity increases circulating blood volume and cardiac output, chronically elevating ventricular wall stress and predisposing to concentric and eccentric remodeling [71]. Insulin resistance and atherogenic dyslipidemia accelerate coronary atherosclerosis and ischemic cardiomyopathy, while ectopic lipid accumulation and adipose-derived cytokines promote lipotoxicity, mitochondrial dysfunction, and impaired myocardial contractility [72,73].

Epidemiological studies highlight the magnitude of this association. In the Framingham Heart Study, obesity independently increased the risk of HF, with a 5% rise in incident HF for each unit increase in BMI [74]. Similarly, the Atherosclerosis Risk in Communities (ARIC) study demonstrated that the presence of MetS doubled the risk of HF compared to metabolically healthy individuals [74]. Obesity and insulin resistance are particularly linked to HF with preserved ejection fraction (HFpEF), characterized by diastolic dysfunction, systemic inflammation, and microvascular rarefaction [75]. By contrast, MetS also contributes to HF with reduced ejection fraction (HFrEF), predominantly through accelerated atherosclerosis and neurohormonal activation [74,76]. Importantly, visceral adiposity and insulin resistance predict HF more strongly than overall adiposity, underscoring the metabolic rather than purely mechanical burden of obesity [77].

Pathophysiologically, adipose tissue expansion enhances the activation of RAAS and SNS, both of which promote hypertrophy, fibrosis, and impaired relaxation [40]. Systemic low-grade inflammation exacerbates endothelial dysfunction, arterial stiffness, and microvascular dysfunction, key drivers of HFpEF progression [75]. Together, these pathways create a maladaptive cardiometabolic environment that sustains the transition from MetS to clinical HF.

From a therapeutic standpoint, neurohormonal blockade remains the foundation of treatment in HFrEF, with ACEIs, ARBs, beta-blockers, MRAs, and angiotensin receptor-neprilysin inhibitors (ARNIs) such as sacubitril/valsartan demonstrating substantial reductions in morbidity and mortality, as evidenced in the PARADIGM-HF trial [77]. In HFpEF, conventional approaches have historically shown limited efficacy; however, recent trials such as EMPEROR-Preserved and DELIVER established that SGLT2 inhibitors significantly reduce HF hospitalizations and cardiovascular death, irrespective of diabetes status, marking a paradigm shift in the management of cardiometabolic HF [78,79].

Altogether, HF in the context of obesity and MetS is not merely a hemodynamic disorder but a cardiometabolic disease shaped by neurohormonal, inflammatory, and metabolic derangements. This recognition emphasizes the need for integrated therapeutic strategies that extend beyond blood pressure and volume control to target the underlying metabolic-inflammatory milieu, thereby redefining the clinical approach to HF prevention and treatment.

Non-alcoholic Fatty Liver Disease (NAFLD) and Its Cardiovascular Implications

The hepatic condition known as NAFLD now operates under the name MASLD because it represents the liver manifestation of MetS and has become a leading risk factor for CVD. The condition NAFLD affects 25-30% of people worldwide, while obesity and insulin resistance and type 2 diabetes increase the risk of developing ASCVD [80,81]. A comparative overview of lifestyle and pharmacological interventions and their effects on lipid fractions and cardiovascular outcomes, as an integration between NAFLD and atherogenic dyslipidemia, is summarized in Table 1.

**Table 1:** Effects of lifestyle and pharmacological interventions on atherogenic dyslipidemia and cardiometabolic risk in the context of NAFLD This table presents how major lifestyle changes and pharmacological treatments affect LDL-C, TG, HDL-C, and sd-LDL levels and their impact on cardiovascular health. The different interventions show a complete therapeutic method to treat atherogenic dyslipidemia and prevent atherosclerotic cardiovascular disease progression. The arrows indicate the direction and magnitude of the effect of each intervention on lipid levels and cardiovascular outcomes: ↓ represents a mild or modest reduction; ↓↓ represents a moderate reduction; ↓↓↓ represents a strong reduction; ↑ represents a slight increase; ↔ suggests no significant change or effect. LDL-C: low-density lipoprotein cholesterol; TG: triglycerides; HDL-C: high-density lipoprotein cholesterol; sd-LDL: small dense low-density lipoprotein; ASCVD: atherosclerotic cardiovascular disease; CV: cardiovascular; EPA: eicosapentaenoic acid; DHA: docosahexaenoic acid; HF: heart failure; MACE: major adverse cardiovascular events; ANGPTL3: angiopoietin-like 3; REDUCE-IT: Reduction of Cardiovascular Events with Icosapent Ethyl-Intervention Trial

Intervention	Effect on LDL-C	Effect on TG	Effect on HDL-C	Effect on sd-LDL	Cardiovascular outcomes	Key references
Lifestyle (diet+exercise)	↓ (modest)	↓	↑	↓	Improved overall risk	[4,14]
Statins	↓↓↓	↓ (mild)	↔/↑	↔	Strong reduction in ASCVD	[24]
PCSK9 inhibitors	↓↓↓	↔	↔	↔	Significant ASCVD reduction	[24]
Fibrates	↓ (mild)	↓↓	↑	↓	Limited outcome benefit	[23]
Omega-3 fatty acids (EPA/DHA)	↔	↓↓	↑	↓	REDUCE-IT: ↓ CV events (EPA only)	[25]
ApoC-III/ANGPTL3 inhibitors	↔	↓↓↓	↑	↓	Emerging data, promising	[26]
GLP-1 receptor agonists	↓ (mild)	↓ (mild)	↑	↔	↓ MACE	[53]
SGLT2 inhibitors	↓ (mild)	↓ (mild)	↔	↔	↓ HF hospitalization, ↓ CV death	[81]

**Figure 1:**
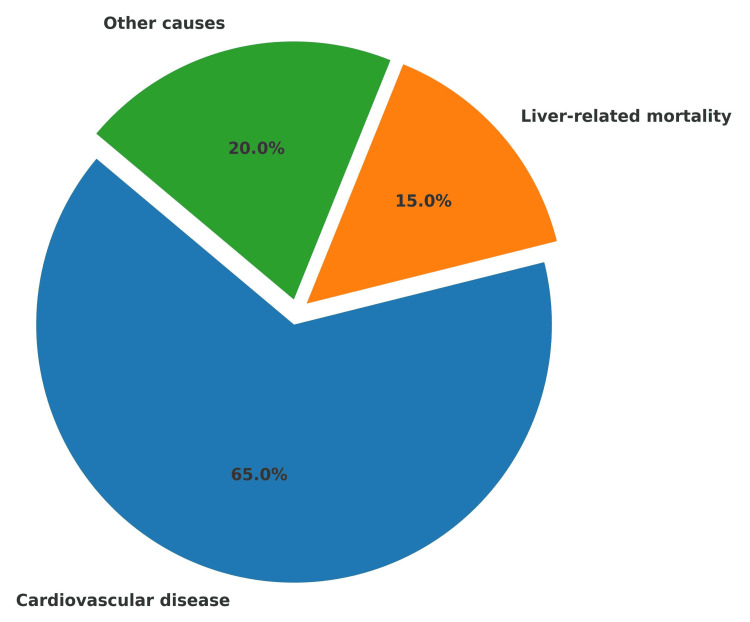
Causes of mortality in patients with NAFLD/MASLD Distribution of mortality in NAFLD, recently redefined as MASLD. Cardiovascular disease represents the leading cause of death (~65%), followed by liver-related mortality (~15%) and other causes (~20%) [82,83]. NAFLD: non-alcoholic fatty liver disease; MASLD: metabolic dysfunction-associated steatotic liver disease

The main cause of death in NAFLD patients is CVD according to epidemiological studies which show that liver failure complications like cirrhosis and hepatocellular carcinoma are less significant [82]. As illustrated in Figure 1, CVD accounts for the majority of deaths among patients with NAFLD/MASLD, far exceeding liver-related mortality and other causes.

**Figure 2:**
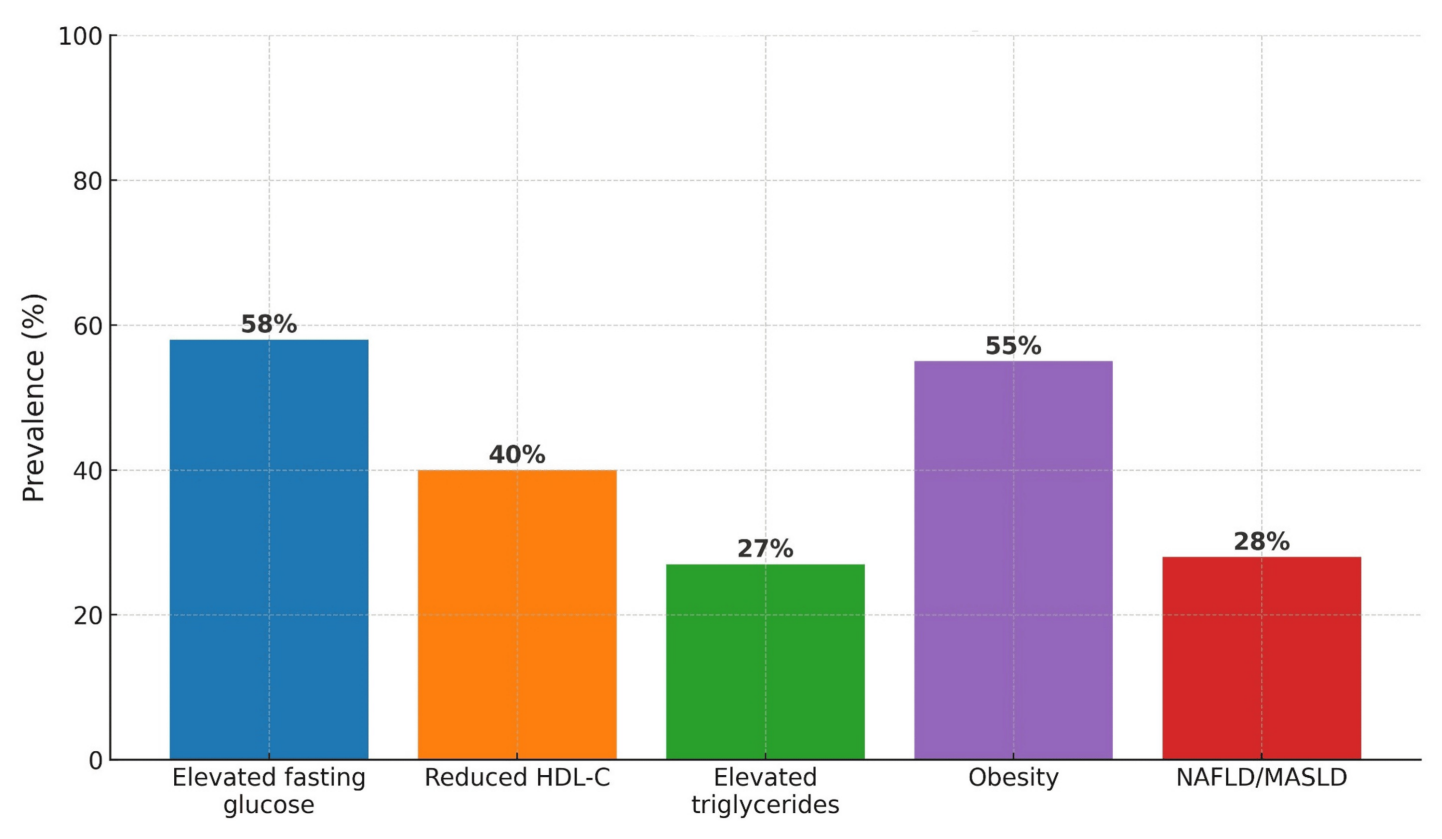
Prevalence of metabolic syndrome components and NAFLD/MASLD Bar chart illustrating the prevalence of major metabolic syndrome abnormalities in US adults (NHANES 1999-2018) and the global burden of NAFLD/MASLD. Elevated fasting glucose (~58%) and obesity (~55%) are the most common abnormalities, followed by reduced HDL-C (~40%), elevated triglycerides (~27%), and NAFLD/MASLD (~28%). Data derived from Younossi et al. [80] and Browning et al. [89]. NAFLD: non-alcoholic fatty liver disease; MASLD: metabolic dysfunction-associated steatotic liver disease; NHANES: National Health and Nutrition Examination Survey; HDL-C: high-density lipoprotein cholesterol

Multiple elements in the body create the link between NAFLD and CVD. The combination of hepatic steatosis and inflammation creates body-wide insulin resistance and lipid disorders which produce harmful lipoproteins that produce additional small dense LDL particles and triglyceride-rich remnants [84]. The inflamed fatty liver produces pro-inflammatory cytokines (IL-6, TNF-α) and prothrombotic factors while decreasing hepatokines fetuin-A and adiponectin which leads to increased systemic inflammation and endothelial dysfunction and atherogenesis [85,86]. The progression of NAFLD to non-alcoholic steatohepatitis (NASH) and fibrosis leads to coronary microvascular dysfunction and increased arterial stiffness and left ventricular diastolic dysfunction which indicates that liver disease directly causes cardiomyopathy [87,88]. Figure 2 illustrates how NAFLD/MASLD prevalence correlates with the prevalence of MetS components in US adults. 

The large-scale research design of cohort studies provides a strong basis for medical evidence development. The Dallas Heart Study found that NAFLD independently caused subclinical atherosclerosis through coronary artery calcium measurements after adjusting for known risk factors [10]. The research included 34,000 participants to show that NAFLD patients face a 45% higher risk of fatal and nonfatal cardiovascular events than people without NAFLD [10]. The severity of fibrosis stands as the primary risk factor for cardiovascular complications rather than the presence of steatosis alone according to research [90]. 

The main therapeutic approaches for NAFLD patients with cardiovascular risks involve lifestyle changes and weight reduction because these interventions enhance both liver health and heart health. The available pharmacological treatments are restricted, but GLP-1 receptor agonists and SGLT2 inhibitors have demonstrated effectiveness in clinical trials by lowering liver fat and enhancing heart and metabolic health [91,92]. Research into new drugs that focus on fibrosis and inflammation and lipid metabolism continues to develop as scientists work to stop liver disease progression while reducing heart disease risk. 

The combination of NAFLD creates a condition that affects the liver and produces systemic effects on cardiometabolic health. The strong independent link between NAFLD and CVD complications requires healthcare providers to develop treatment plans which combine liver disease management with metabolic risk factor control and cardiovascular protection. 

Global policy and public health response

International Strategies and Guidelines

The worldwide prevalence of noncommunicable diseases (NCDs), including obesity, has experienced a significant incidence considering that childhood obesity now represents a major public health concern which compromises physical health, social development, and mental health while leading to obesity in adulthood and increases the probability of developing conditions such as diabetes and CVD [93-96]. In 2014, an estimated 41 million children under five years were classified as overweight or obese worldwide, with rates nearly doubling in Africa from 5.4 million in 1990 to 10.3 million in 2014. The data show a more rapid increase in low- and middle-income countries than in high-income ones, reflecting a shift in the obesogenic environment driven by globalization, urbanization, changes in food availability, and physical activity [94,95]. This epidemic is driven by energy imbalance from increased consumption of ultra-processed, energy-dense, nutrient-poor foods and sugar-sweetened beverages (SSBs) coupled with reduced physical activity; in addition, there is an inaccurate cultural understanding, in some places, that an overweight infant is considered a proxy of healthy development [94-97]. Considering this, the global responses and strategies need to cover an individual's entire lifespan, preventing bad lifestyles and risk factors frequently seen and adopted from the social environments [93-96].

The WHO has created detailed worldwide strategies for NCD prevention through the Global Action Plan for the Prevention and Control of NCDs 2013-2020 which now runs until 2030 to support Sustainable Development Goals (SDGs) [93,96]. The Global Action Plan for the Prevention and Control of Noncommunicable Diseases (NCDs) outlines six interdependent objectives which are the following: to raise the priority accorded to NCD prevention and control through strengthened international cooperation and advocacy; to strengthen national capacity, leadership, governance, multisectoral action, and partnerships to accelerate country responses; to reduce modifiable risk factors and underlying social determinants by creating health-promoting environments; to strengthen and orient health systems to address NCD prevention and control through people-centered primary healthcare and universal health coverage; to promote and support national capacity for high-quality research and development; and to monitor trends and determinants of NCDs while evaluating progress in their prevention and control. Key voluntary global targets include a 25% relative reduction in premature mortality from NCDs by 2025 extending to a one-third reduction by 2030, preventing the rise in diabetes and obesity prevalence, and specific reductions in risk factors such as a 30% decrease in salt/sodium intake by 2025 and 40% by 2030, a 30% reduction in tobacco use by 2025 and 40% by 2030, and a 10% decrease in harmful alcohol use by 2025 and 20% by 2030 [93-96].** **The Pan American Health Organization (PAHO) Plan of Action for the Prevention of Obesity in Children and Adolescents (2014-2019) serves as a complementary regional strategy to stop obesity growth in the Americas through primary healthcare and school environment improvements, fiscal policy changes, and multisectoral cooperation for managing the dual nutrition problem of undernutrition and obesity [94,95]. The strategies promote whole-of-government methods which unite education with agriculture and trade and urban planning to establish supportive environments for healthier eating habits and physical exercise while focusing on equal opportunities for social minorities such as indigenous and migrant children [93-97].

The international guidelines establish food marketing restrictions for children as research proves that children who are exposed to advertisements for high-fat, high-sugar, and high-salt foods develop stronger food preferences which leads to higher purchasing demands and increased food consumption rates that result in obesity [95-97]. The WHO Set of Recommendations on the Marketing of Foods and Non-alcoholic Beverages to Children advises Member States to establish full policies which restrict food marketing on television and the internet and in schools to reduce child exposure to persuasive advertisements while promoting global cooperation for managing international advertising [95-97]. The PAHO uses fiscal strategies to address childhood obesity through SSBs and energy-dense/nutrient-poor product taxation as demonstrated by Mexico's excise tax which decreased SSB sales to decrease consumption and promote healthier nutritional habits. Supported by public education and nutrient profiling, the implementation of interpretive front-of-package labeling systems enables caregivers and children to make informed choices about foods high in sugars, fats, or salt. As supportive initiatives to foster supportive health-promoting environments in schools, the restrictions on the provision, sale, and marketing of unhealthy foods and beverages including standards for meals that align with healthy nutrition guidelines and ensuring access to potable water are excellent measures to address childhood obesity [95-97].** **The Commission on Ending Childhood Obesity supports these recommendations through the demand for nutrient profiling to identify unhealthy foods, standardized labeling, and restrictions in child-gathering settings as voluntary industry codes fail to protect all children effectively [95-97].

The prevention of obesity depends on two essential components: breastfeeding promotion and early childhood nutrition. A 10-20% reduction in the risk of childhood obesity is associated with exclusive breastfeeding for the first six months of life as it promotes healthy growth patterns and appropriate weight gain during infancy [94,95].** **The guidelines for complementary feeding promote the avoidance of SSBs and energy-dense foods to help children develop proper eating habits [94,95]. The Global Action Plan and PAHO's plan incorporate these recommendations into preconception and antenatal care to address maternal factors that increase the risk of obesity in offspring through epigenetic changes by advising weight management, nutritional counseling, and toxin avoidance for expectant parents [93-96]. The guidelines for physical activity recommend at least 60 minutes of moderate-to-vigorous exercise each day which should be integrated into school programs and public areas to fight sedentary behavior, often as a consequence of prolonged screen use, through combined multisectoral efforts that create secure cities with accessible facilities for all genders [94-96].

The WHO monitors BMI for age as an NCD indicator through its Global Monitoring Framework for 2030, while PAHO executes regional plans that use disaggregated data through equity stratifiers to minimize health inequalities [1,3,5]. The Commission needs strong systems to track obesogenic environments, life-course risks, and intervention success rates through existing frameworks such as the Global School-based Student Health Survey to establish evidence-based context-specific methods and financial effects [93,95,96]. The implementation of targets receives oversight through accountability frameworks which use civil society organizations and private sector entities to create transparent progress reports [93-96]. The main objective of these international strategies aims to stop childhood obesity growth from 2025 to 2030 while working to decrease NCD premature deaths by one-third and create health equity through lifespan interventions that fight unhealthy eating and sedentary behavior to build settings which support child development and break obesity patterns between generations and achieve SDG targets for sustainable development [93-97].

Fiscal Policies: Sugar Taxes and Food Subsidies

The WHO, together with PAHO, supports sugar taxes and food subsidies as essential financial tools to address the issue of obesity and cardiometabolic diseases worldwide [98-100]. The policy framework consists of two main elements which include excise taxes on SSBs and non-essential energy-dense foods (NEDFs) with varying sugar content to promote product reformulation and provide financial assistance to low-income families through subsidies for healthy food items, for instance, fruits, vegetables, and water [98-101]. The 2014 Mexican government implemented a one-peso-per-liter excise tax on sugary beverages which amounts to 10% of the product value while imposing an 8% ad valorem tax on NEDFs with more than 275 kcal/100 g content. The tax system emerged from a bipartisan legislative effort that used Article 4 of the Constitution to establish public health protection as a state responsibility [100,102-105]. The Spanish region of Catalonia implemented a tiered excise tax system for SSBs in 2017 which followed WHO guidelines by setting higher tax rates for beverages with higher sugar content [98-101]. Research based on real-world SSB tax evaluations across multiple jurisdictions, including those mentioned, shows that a 10% tax equivalent leads to a 10% decrease in SSB consumption and dietary intake. The research shows that SSB tax effects become more pronounced with time and differ based on socioeconomic status (SES) [101,103,106].

Studies based on post-implementation evaluations show that sugar taxes continue to affect consumer behavior by decreasing SSB consumption while people choose alternative beverages that are either tax-free or healthier, but subsidies would enhance these changes [98-101,103,106]. Analyzed data from Mexico's 2012-2015 household purchase records revealed an 8.2% decrease in taxed SSB sales during the two-year period after the tax implementation. The data showed that taxed SSB purchases decreased by 5.5% in the first year and dropped by 9.7% in the second year after the tax introduction. This policy demonstrated progressive effects because it reduced SSB purchases most among low-income households even though some experts had predicted regressive effects [100,103,105]. This aligns with Catalonia's experience, where the tax led to a 16.7% per capita decline in SSB purchases three and a half years post-implementation, with reductions progressively increasing from 10.4% after one year to 15.3% at three years, accompanied by a 21.7% rise in non-sugar-sweetened beverages (NSSBs) but no change in bottled water consumption [101]. The modeling studies show that SSB taxation will lead to better health outcomes in the future because Mexico's 10% reduction in consumption would stop 189,300 new diabetes cases and 20,400 CVD events and 18,900 deaths from occurring within 10 years. The healthcare cost savings would total US$983 million, while the most significant benefits would go to low SES groups and young adults when SSB consumption drops by 20% [105]. The implementation of financial incentives as de facto subsidies for healthy behaviors proved to have a moderate success in promoting physical activity through short-term effects. The implementation of a US$1.19 daily incentive led to a 0.52 standardized mean difference increase in daily steps, but this effect reduced to 0.20 during follow-up, thus requiring food subsidy integration to maintain obesity prevention [107]. The WHO supports using SSB taxes along with healthy food subsidies as research shows that food subsidies lead to 10-15% higher fruit and vegetable consumption while SSB taxes decrease their sales by 10-20% and suggests using tax money for health promotion to address equity gaps in low- and middle-income countries [98-100].

The implementation of these fiscal measures includes strategies to fight industry resistance while achieving public health benefits through product reformulation incentives and revenue allocation for promoting healthier options as demonstrated by Mexico's NEDF tax framework [98-104]. The 8% NEDF tax in Mexico for children will lead to a 5.1% reduction in caloric content of taxed products, consequently leading to a BMI reduction of 0.08-0.13 units and a 1.6-2.4% decrease in overweight and obesity rates among 6-17-year-olds with more pronounced effects in boys, urban residents, and higher SES groups. The policy serves as a preventive measure against cardiometabolic risks that can be passed from one generation to the next [104]. The pediatric focus matches global meta-analyses which demonstrate that tax effects differ substantially (I²=97%) because SSB consumption decreases but water consumption shows no significant change increasing 1.9% which indicates that specific subsidies could enhance the substitution effect [9]. The price pass-through for the tax in Catalonia reached 11% (0.11 €/L) with full transmission to regular soda prices but only partial transmission to energy drinks according to WHO technical reports recommending continuous surveillance to optimize policy adjustments [99,101]. PAHO's documentation of Mexico's tax highlights pre-tax SSB consumption at 163 liters per person annually, with early evaluations showing a 10% drop, and stresses the importance of multisectoral collaboration to integrate subsidies for traditional, nutrient-rich foods amid the double burden of malnutrition in low- and middle-income countries [100]. Research on the financial incentives for physical activity through a meta-analysis of $1.19 USD daily rewards established a moderate effect on daily step counts above 1,000 steps but found no lasting benefits after the incentive programs stopped; results depended on the incentive amount and duration and device type and goal establishment methods, but there is insufficient long-term data to confirm the relationship [107]. 

The implementation of sugar taxes in low- and middle-income countries would produce significant revenue streams potentially reaching trillions of dollars over 15 years for health system development and subsidy programs, yet requires international cooperation to address trade barriers and industry opposition [98-100,105]. The Mexican legislative process established the tax through constitutional means to fight obesity-related social expenses while receiving support from 37 members across different political parties which demonstrates how health rights can make taxation politically viable [5]. The absolute tax regressive nature is mild, yet the health benefits of taxation create more advantages for low SES groups due to larger consumption decreases and higher NCD aversions [103,105,106]. The long-term effects of Catalonia during the COVID-19 pandemic demonstrate resilience in light of the fact that NSSBs and water prices remained stable, yet they can be improved through subsidy programs [101]. The WHO holds meetings focused on the logical basis for implementing fiscal policies to address market failures that result from cheap and accessible unhealthy food options in obesogenic environments. Research from more than 50 nations demonstrates that SSB consumption decreases when the tax range is between 10% and 30% without incurring significant market disruptions [99]. The modeling evidence shows that a 0.5% BMI reduction would occur in Mexico due to the SSB tax, while the combination of the tax with food price subsidies ranging from 10% to 25% would yield more substantial effects [98,99,105].

The fiscal policies collaborate to achieve dual objectives that cover decreasing SSB and NEDF consumption by 10-20% through taxation while preventing between 200,000 and 400,000 diabetes and CVD new cases, reducing childhood obesity rates by 1.6-2.4% and reducing NCD premature deaths by up to one-third by 2030. The subsidy program works to increase healthy food and physical activity consumption by 10-25% which will create sustainable dietary changes and generate funds for health promotion initiatives in low- and middle-income countries [98-107].

Front-of-Pack Labelling (FOPL) and Marketing Regulations

The WHO, PAHO, and Food and Agriculture Organization (FAO) have established frameworks for implementing FOPL and marketing regulations which serve as vital public health measures to fight obesity and cardiometabolic diseases by helping consumers understand nutritional values and preventing deceptive marketing of unhealthy foods [108-111]. The systems use interpretive schemes to present octagonal warning labels for excess sugar, salt, saturated fats, trans fats, and calories to help consumers understand complex nutrition data on packaging labels; this approach influences both consumer behavior and product reformulation to stop misleading promotions that target children and people from low-income backgrounds [108-116]. The Americas have seen rapid adoption of FOPL because all 35 countries studied the policy and 30 countries created proposals while 11 countries adopted policies and seven countries (Argentina, Chile, Ecuador, Mexico, Peru, Uruguay, and Venezuela) made FOPL mandatory by 2022; the seven countries adopted black octagon labels which use "excess" instead of "high in" to enhance effectiveness while following PAHO's nutrient profile model for defining strict nutritional limits [108,111,115]. Research indicates that FOPL schemes which use warning labels, for specific nutrients and traffic light systems, perform better than summary icons and health checks in actual retail environments because they lead to a 10-30% decrease in unhealthy food and beverage sales and better nutritional comprehension among all consumer groups. The schemes demonstrate lower success rates in controlled experimental tests where effects are sometimes less pronounced [110,112,114,116].

Studies where FOPL systems were implemented, as empirical evidence, demonstrate that these systems lead to lower consumption of unhealthy foods while marketing restrictions through celebrity endorsements and child-directed claims achieve high levels of industry compliance and product reformulation [108,110-113,115]. The 2020 NOM-051 update in Mexico brought octagonal warning labels that restrict health and nutrition claims on 80.8% of packaged items with high energy and sugar and sodium and saturated fat and trans fat content which researchers predict will reduce such claims by 36.8% in total and by 61.1% in ultra-processed foods according to a study of 17,264 products that showed pre-regulation claim rates at 33.8% for nutrition claims and 3.4% for health claims [113,115]. The warning labels introduced in 2016 by Chile became a model for other countries because they reached 90% compliance and led to a 23-24% decrease in SSB sales and a 7% reduction in sugar content through product reformulation and stronger effects were observed in low SES households where label comprehension and application rose by 20-30% [108,110-112]. The Food and Drug Administration (FDA) conducted quantitative research across the United States using experimental designs with more than 5,000 participants to evaluate different FOPL schemes which demonstrated that interpretive labels such as nutrient warnings and traffic lights increased healthier choices by 15-25% when used with claim restrictions but power analysis showed that bigger participant numbers were required to identify subtle SES variations [116]. Broader systematic reviews from 2011 to 2022, encompassing 32 studies across diverse populations, confirm that FOPL enhances objective understanding (e.g., identifying healthier options) and subjective comprehension (e.g., perceived healthiness), with low SES groups benefiting most from simple warning formats that mitigate marketing influences, yet gaps remain in long-term behavioral change and equity-focused evaluations [110,114].

FOPL regulations in Latin America and the Caribbean (LAC) have evolved to become more protective through the implementation of black contrasting backgrounds for better visibility and the prohibition of cartoon marketing on food labels which together reduce children's exposure to unhealthy food promotions and promote better eating habits [108,109,111,115]. The sodium and sugar reduction in processed foods of Peru and Uruguay follows Chile's model, while Argentina adopted a child protection approach through warning label bans on products with health warnings in 2022 which follows WHO recommendations for evidence-based mandatory FOPL to fight against obesogenic marketing environments [108,109,111]. The FOPL interventions conducted in controlled virtual supermarkets or lab stores resulted in a 10-20% reduction of unhealthy purchases, but real-world retail studies in Ecuador and Venezuela achieved better results through proper labeling standards which led to a 30% decrease in high-sugar product sales [108,110,112]. The PAHO/FAO/United Nations Children's Fund (UNICEF) guidance notes for LAC explain that FOPL combined with marketing bans fights the dual problem of malnutrition because 49.8% of Mexican products needed warnings before the regulation and recommends using PAHO's nutrient profiles to establish safety thresholds which protect against diabetes and hypertension [4,6]. The FDA conducted a literature review from 2005 to 2022 of more than 50 systematic analyses to determine that warning labels work best for low SES consumers who benefit from these labels to fight against targeted marketing but need continuous policy adjustments to address label clutter and consumer fatigue [110,116].

FOPL frameworks contain marketing regulations which limit promotional methods and allow health claims only on products that meet nutrient standards to decrease ultra-processed food appeal and boost whole food sales [108,109,111,113,115]. The NOM-051 in Mexico prohibits all forms of endorsements, interactive content, and free gifts on warning labels which results in a 36.8% decrease in claims that primarily affects beverage products (61.1% ultra-processed decline), and high-in products in Chile experience a 20-30% reduction in child-directed advertising [108,113,115]. The WHO framework manual supports FOPL principles through its requirement for independence from industry influence and dietary guideline alignment because NCDs result in 41 million yearly deaths worldwide and diet quality emerges as the main cause of these deaths while it sets up tracking systems to check marketing compliance [112]. The experimental FDA research that combined screenshots with questionnaires demonstrated FOPL schemes with warning labels made participants from low SES backgrounds more aware of cardiometabolic disease risks, but they needed back-of-pack information to understand the complete extent of risks [110,114,116]. The Americas region shows that Ecuador and other early adopters encountered industry opposition at first but reached high compliance levels through step-by-step implementation of reformulation which reduced trans fat content by 10%, thus demonstrating how FOPL-linked marketing bans can transform food supply systems [108,111].

The main goals of FOPL and marketing regulations are to decrease unhealthy food buying by 10-30% which will prevent millions of NCD cases. The initiative aims to boost consumer knowledge and application of food labels across different socioeconomic levels by 20-30% while maintaining equal attention to low-income populations; this will drive industry changes to reduce sugar content, salt, and fat levels by 7-10% while banning deceptive labeling on 80.8% of non-compliant products to create better health environments in order to achieve more than 90% compliance rates while creating enduring dietary changes throughout Latin America and worldwide [108-116].

Promoting Healthy Lifestyles: Urban Planning and Physical Activity

Urban planning serves as a fundamental component for creating public spaces which promote physical activity as it aids in the fight against obesity and MetS by building accessible safe areas that encourage exercise [117-126]. Systematic reviews demonstrate that built environment features such as walkable neighborhoods, accessible green spaces, and active transportation infrastructure lead to higher physical activity levels among different population groups [117-119,121,123,126]. Research shows that people who reside within a 20-minute walking radius from parks, playgrounds, and sports facilities achieve superior physical activity outcomes (OR 1.20-1.25) because these facilities enable both functional and recreational activities in urban environments [118,122]. The findings from prospective cohort studies indicate that city residents who use active transportation through designated cycling paths and walking routes have decreased risks of CVD (HR 0.54 for cycling) and total mortality (HR 0.59) which supports the requirement for urban policies that promote walking and cycling to mitigate sedentary behavior [120,123,124,126]. The current socioeconomic position (SEP) differences make it harder for people from lower SEP groups to access safe recreational spaces, limiting their leisure-time physical activity and leading to higher obesity rates. The resolution to these disparities requires fair urban planning, to bridge these gaps and to guarantee inclusive health benefits for the community [119,121,122]. 

Urban planners use green space integration as their main approach as research shows that people who live in the most verdant areas achieve 27% higher physical activity levels than those in the least green areas [118,121,123]. The connection between cycling and health benefits reaches past recreational activities because it shapes daily routines and leads to decreased carbon emissions and cleaner air which indirectly benefits heart and metabolic health [118,120,121,124]. Economic studies show that bicycle infrastructure investments between $28 and $69 million CAD in particular cities generate 1.7:1 to 4.9:1 benefit-cost ratios during a 10-year period, as they prevent 9-43 premature deaths and cardiometabolic events, thus proving the economic value of urban spaces that support walking and cycling [124]. However, challenges persist in regions like Latin America, where only 14.5% of adults consider their neighborhoods suitable for walking and cycling; nevertheless, 39.75% of residents report safety concerns, necessitating immediate urban development to improve both accessibility and security [122,123,126]. The global frameworks support multisectoral approaches which create active communities through urban regulations that mandate green corridors and mixed-use development to promote physical activity, protect biodiversity, and urban cooling [121,123,125,126]. 

Systematic reviews together with policy documents show that developing urban planning strategies to reduce obesity prevalence requires robust research methods like longitudinal studies and complex statistical models that use moderators (e.g., ethnicity, age) and mediators (e.g., perceived safety) to establish causality [117,119,121,123]. The implementation of counseling for active lifestyles in primary healthcare facilities becomes more effective through urban community transformations, establishing environments that promote preventive measures against NCDs [125]. Participatory urban agendas require all stakeholders, including government entities and civil society organizations, to work together to redesign cities with accessible metropolitan parks and integrated public transportation systems which research indicates decrease CVD mortality rates by 48-64% and enhance social sense of belonging [117,118,120,121,126]. The economic evaluations show that these benefits remain stable when using different time frames and statistical life value assessments yet demonstrate that proper urban infrastructure planning leads to major health benefits including 87000-349000 tonnes of carbon emission reduction during a 10-year period [124]. 

Urban planning initiatives work to achieve three main goals: reducing global physical inactivity to 15% below 2030 targets through active environment development and safe transportation systems, accessible recreational facilities to prevent 9-43 premature deaths per urban investment cycle, and reducing cardiometabolic conditions such as obesity and CVD by promoting daily physical activity at WHO-recommended levels [1-10]. The goals require multisectoral work for solving safety perception issues and economic inequalities through cost-efficient strategies and support sustainable development to reduce NCD healthcare expenses by INT$54 billion yearly and build climate-resistant urban health systems [119-124,126].

## Conclusions

Obesity and MetS have emerged as a global pandemic, driving an alarming rise in cardiovascular morbidity and mortality. As outlined in this review, insulin resistance, atherogenic dyslipidemia, systemic inflammation, endothelial dysfunction, and neurohormonal activation act synergistically as an interconnected cardiometabolic network to accelerate the onset of CAD, stroke, HF, and NAFLD. Structural public health interventions such as SSB taxation, FOPL, and marketing restrictions have demonstrated measurable reductions in the consumption of unhealthy products and improvements in population-level health behaviors. In conclusion, the escalating prevalence of obesity and MetS demands a paradigm shift that integrates the biological, behavioral, and societal determinants of the disease. Through coordinated action that unites clinical innovation with evidence-based public health policies, it is possible to alter the trajectory of CVD in the 21st century and secure healthier futures for the upcoming generations.
